# Cognitive and neuroanatomical impairments associated with chronic exposure to levamisole-contaminated cocaine

**DOI:** 10.1038/s41398-018-0279-3

**Published:** 2018-10-27

**Authors:** Matthias Vonmoos, Sarah Hirsiger, Katrin H. Preller, Lea M. Hulka, Daniel Allemann, Marcus Herdener, Markus R. Baumgartner, Boris B. Quednow

**Affiliations:** 1 Department of Psychiatry, Psychotherapy, and Psychosomatics, Psychiatric Hospital, Experimental and Clinical Pharmacopsychology, University of Zurich, Zurich, Switzerland; 2 Department of Psychiatry, Psychotherapy, and Psychosomatics, Psychiatric Hospital, Center for Addictive Disorders, University of Zurich, Zurich, Switzerland; 3 Health & Social Welfare Department State of Berne, Office of the Cantonal Pharmacist, Zurich, Switzerland; 4 Center of Forensic Hairanalytics, Institute of Forensic Medicine, University of Zurich, Zurich, Switzerland; 5 Neuroscience Center Zurich, University of Zurich and Swiss Federal Institute of Technology Zurich, Zurich, Switzerland

## Abstract

Currently, levamisole is the most common cocaine adulterant worldwide and it is known to induce a variety of adverse side effects. Animal studies and human case reports suggest potential neurotoxicity of the compound but neither neuroanatomical nor cognitive effects of levamisole have been systematically investigated in cocaine users so far. We examined cognitive performance and cortical structural differences between chronic cocaine users with low and high recent exposure to levamisole objectively determined by quantitative toxicological hair analyses. In Study 1, we compared 26 chronic cocaine users with low levamisole exposure (lowLevCU), 49 matched cocaine users with high levamisole exposure (highLevCU), and 78 matched stimulant-naive controls regarding cognitive functioning employing a comprehensive neuropsychological test battery. In Study 2, we investigated cortical thickness by use of T1-weighted MRI in a subgroup of 12 lowLevCU, 17 highLevCU, and 38 stimulant-naive controls. In Study 1, both cocaine user groups showed significant impairments in the cognitive domains of attention and working memory as well as in the global cognitive index. However, highLevCU showed significantly worse executive functions compared to lowLevCU although both groups did not differ in severity of cocaine consumption and other clinical dimensions. Study 2 revealed that highLevCU, displayed reduced cortical thickness specifically in the middle frontal gyrus compared to both controls and lowLevCU. Our results suggest that levamisole exposure during the last months in cocaine users is associated with increased executive function impairments and pronounced thinning of the lateral prefrontal cortex. Consequently, prevention and drug policy-making should aim to reduce levamisole contamination of street cocaine.

## Introduction

**Fig. 1:**
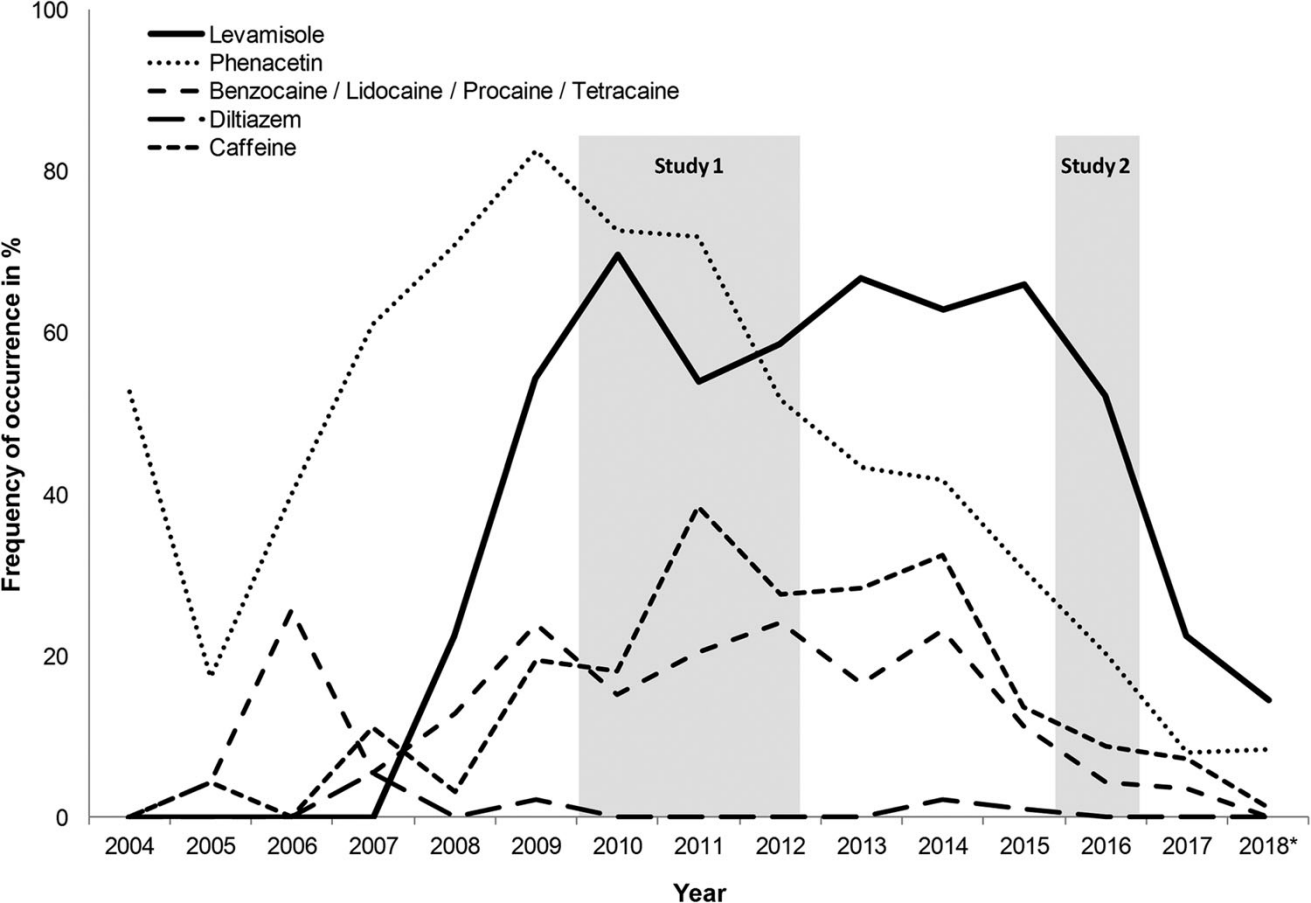
Additives in cocaine samples in Switzerland between 2004 and 2018. Lines indicate percent frequency of occurrence. Recruitment periods of cocaine users for both studies are shaded in gray. The data were collected in mobile laboratories in Berne, Zurich, and Basel (total *n* = 771). Data were provided by the Office of the Cantonal Pharmacist, Health & Social Welfare Department State of Berne, Switzerland (Daniel Allemann, Hans-Jörg Helmlin, and André Mürner). *Data only from the first half-year 2018 (January–August)

The tetramisole enantiomer levamisole is used as a veterinary anthelminthic that was also approved as an adjuvant in colon cancer treatment in some countries before it was withdrawn from the market in 2000 because of its adverse side effects^1^. In 2004, the U.S. Drug Enforcement Agency (DEA) initially detected levamisole as a adulterant in cocaine seizures^2^. In the mobile drug-checking program of Switzerland, levamisole was recognized in 2008 for the first time as an adulterant in street cocaine. Measurements revealed that between 2009 and 2016, 50 to 70% of all cocaine specimens contained levamisole (Fig. 1). Similar trends of extensive levamisole contamination of street cocaine across the last decade were shown for the US and for different European countries^3,4^. The recent drop of the levamisole prevalence in Switzerland is a phenomenon that to our knowledge has not been shown in other countries so far (Fig. 1). By contrast, in October 2017, the DEA reported that 87% of the seized and analyzed cocaine bricks contained levamisole.^4^ Thus, levamisole is currently the most common cocaine adulterant in Europe and North America^3,4^.

The mixture of cocaine with other pharmacological components (primarily prescription drugs and over-the-counter agents, see Fig. 1) prior to being sold on the streets lead to a decline of cocaine purity in the main consumer markets of North America and Europe^5^. These adulterants were generally added for two reasons. First, they are available, cheap, have similar chemo-physical properties (color, texture, melting point) and, thus, increase the profit of the drug dealer. Second, some additives are supposed to enhance the psychoactive effects of the drug by exerting additional pharmacological effects^1,6^. In the case of levamisole, it was shown that the compound itself has negligible effects on monoamine transporters, but it was proposed that the mother compound is metabolized—among others—to aminorex, a psychostimulant agent that shows potent amphetamine-like effects^1,6,7^. A drug discrimination study with rats showed very recently that levamisole in fact potentiates the subjective effects of cocaine when administered concomitantly^8^.

Levamisole has a wide range of adverse side effects. In recent years, an accumulating body of literature described a clear linkage between levamisole-adulterated cocaine use and the occurrence of neutropenia and agranulocytosis, vasculitis, retiform purpura and other forms of skin necrosis, vasculopathy, arthralgia, and leukoencephalopathy^1,9–11^. Its potential neurotoxicity was first reported in dogs experimentally exposed to levamisole showing disseminated perivascular cuffing with mononuclear cells throughout the brain^12^. Since 1992, a number of case reports suggested that the association between the administration of levamisole (in cancer therapy or through cocaine intake) and multifocal inflammatory leukoencephalopathy is also apparent in humans^1,9,13^. In sum and although exact data on the prevalence of toxicity related to levamisole-adulterated cocaine abuse are missing so far^3^, its wide distribution and potential neurotoxicity have been classified a serious public health concern worldwide^11,14^. Although it is important to better understand the specific neuropsychiatric risks associated with levamisole exposure^1^ no case–control study investigating the neuropsychiatric risks of levamisole-contaminated cocaine has been published yet.

Previously, we have shown that the intensity of cocaine intake covaries with cognitive impairments in cocaine users (CU), suggesting that the well-described cognitive deficits in this population are largely drug-induced but also potentially reversible^15–17^. In this context, we now hypothesize that cocaine-related cognitive impairments might not only derive from cocaine itself, but also from its main adulterant levamisole. Thus, in Study 1, we compared two CU groups with similar cocaine use severity but with high (highLevCU) vs. low recent levamisole exposure (lowLevCU) and a matched stimulant-naive control group in their performance in a comprehensive neuropsychological test battery. Low vs. high levamisole exposure was categorized according to a levamisole–cocaine ratio (LCR) in hair samples of the participants. Both compounds were measured by cutting-edge quantitative hair analyses. In line with the above mentioned literature of cocaine-induced cognitive dysfunctions and levamisole-induced neurotoxic effects, we hypothesized that higher levamisole exposure is associated with more severe cognitive dysfunctions.

Based on the findings from Study 1, showing significantly worse executive functions in highLevCU compared to lowLevCU, we subsequently performed a second study with structural magnetic resonance imaging (MRI) in a subsample with similar group classification criteria. In Study 2, we focused on regions-of-interest (ROI) in the frontal lobe—which have been consistently linked to executive function measures used in Study 1^18-21^ as well as on an occipital control region in order to examine whether these levamisole-related cognitive dysfunctions are specifically associated to structural alterations of the frontal cortex. Accordingly, we expected that high levamisole exposure is linked to cortical thinning explicitly in the frontal lobe.

## Materials and Methods

### Participants

#### Study 1

The present data were collected as part of the Zurich Cocaine Cognition Study (ZuCo^2^St). The study included 75 CU, 78 healthy and stimulant-naive healthy controls (for recruitment and selection details see Methods S1). The three groups were matched for age, verbal intelligence, sex, and smoking status. The sample of Study 1 shows a 91% overlap with a sample that was previously published^15^. Exclusion criteria for all participants were an acute or previous neurological disorder or head injury, any clinically significant medical disease, and use of prescription drugs affecting the brain. Additional specific exclusion criteria for both CU groups were the use of opioids, polysubstance use, and any Axis I DSM-IV adult psychiatric disorder—with the exception of cocaine, cannabis, and alcohol abuse; a history of affective disorders (acute major depression was excluded); and attention-deficit hyperactivity disorder (ADHD). Specific exclusion criteria for the control subjects were any current or former Axis I DSM-IV psychiatric disorder and any form of addiction or regular illegal drug use (lifetime > 15 occasions), with the exception of recreational cannabis use. Inclusion criteria for the two user groups were cocaine as primary used illegal drug, cocaine use of >0.5 g per month, and an abstinence duration of <6 months. Before the testing session, participants were asked to abstain from illegal substances for at least 72 h and not to consume alcohol for 24 h. Compliance with these instructions was controlled by urine drug screenings (Methods S2). All participants in both studies provided written informed consent and were compensated for their participation. Both studies were approved by the Cantonal Ethics Committee of Zurich.

#### Study 2

A total of 29 CU and 38 healthy cocaine-naive controls were included in Study 2. A subsample of 17 individuals previously participated in Study 1 (8 controls, 9 CU; for details see Methods S3). Exclusion and inclusion criteria for CU and healthy controls were largely identical to Study 1, apart from that psychiatric medication was allowed in CU in Study 2. Moreover, six participants with alcohol dependence (three in each CU group) and two highLevCU with opioid co-use were included for power reasons. However, the inclusion of these participants did not affect the main results (Tables S1/S2). Participants were mostly right-handed (92.5%) and there was no group difference in handedness (*χ*^2^(2) = 3.85, *p* = 0.15).

### Group classification

When available, 6 cm hair samples were cut from the occiput enabling to objectively estimate drug use and levamisole exposure during the last 6 months. Hair samples were analyzed with liquid chromatography-tandem mass spectrometry (Methods S2). As in Study 1 only 2 of 75 CU (2.7%) and in Study 2 only 1 of 29 CU (3.4%) did not display any traces of levamisole in hair, we decided to compare low vs. high exposure groups. The decisive criterion for the group assignment was a LCR (levamisole concentration/cocaine concentration) higher/lower than 25%. The LCR-cutoff of 25% was equal to the mode value in the right-skewed LCR distribution curve (Figure S1).

#### Study 1

CU were split into 26 CU with a LCR of <25% (low levamisole exposure CU, lowLevCU) and 49 CU with a LCR of >25% (high levamisole exposure CU, highLevCU). For 28 of the 75 CU, <6 cm were available so that at least 3 cm samples were analyzed (3-month drug exposure).

#### Study 2

CU were assigned to either the lowLevCU (*n* = 12) or the highLevCU (*n* = 17) group, respectively. For 10 out of 29 CU, only 3 cm hair samples were available.

### Procedure

Trained psychologists conducted the Structured Clinical Interview (SCID-I) according to DSM-IV^22^. Drug use was assessed with the Interview for Psychotropic Drug Consumption and ADHD symptoms by means of the ADHD self-rating scale (ADHD-SR)^23,24^ The verbal IQ was estimated by a standard German vocabulary test^25^.

### Neuropsychological test battery (Study 1)

The test battery consisted of the Letter Number Sequencing Task (LNST)^26^ a German version of the Rey Auditory Verbal Learning Test (RAVLT)^27^ and four tests from the Cambridge Neuropsychological Test Automated Battery (CANTAB, http://www.cantab.com): Rapid Visual Processing (RVP), Spatial Working Memory (SWM), Intra/Extradimensional Set-Shifting (IED), and Paired Associates Learning (PAL). Analogous to our previous work^15,28^, 15 predefined cognitive test parameters were *z*-transformed on the basis of means and standard deviations of the control group (*n* = 78) and—in respect of data reduction—combined into four cognitive domains (attention, working memory, declarative memory, and executive function). These four domains were further equally integrated into a global cognitive index (GCI)^15,28^.

### Structural MRI acquisition and image processing (Study 2)

All subjects were scanned using a 3T Philips Achieva whole-body scanner equipped with a 32-channel receive head coil. High-resolution structural scans were collected using a standard T1-weighted 3D magnetization-prepared rapid gradient echo (MPRAGE) pulse sequence with repetition time (TR) = 8.08 ms, echo time (TE) = 3.7, field of view (FOV) = 240×240 mm, 160 slices, voxel size of (1×1×1)mm^3^. Cortical surface reconstruction was performed using the software package FreeSurfer v5.3.0 (http://surfer.nmr.mgh.harvard.edu/, Methods S4)^29–31^. ROIs were extracted by parcelating the cortex using the Desikan–Killiany Atlas^32^. Based on the findings from Study 1, we restricted our analysis to ROIs in the lateral frontal lobes. Next to the mean cortical thickness over the whole cortical surface, our analysis included the middle frontal gyrus (MFG, caudal and rostral MFG), inferior frontal gyrus (IFG, pars opercularis, pars orbitalis, pars triangularis), and the lateral orbitofrontal gyrus (lOFG). We also included the superior frontal gyrus (SFG), a region associated with executive functions^33^. The pericalcarine cortex (primary visual cortex) was used as a control region due to its low concentration of dopamine transporters^34,35^ and low involvement in executive functions (Figure S2). As we did not expect lateralized effects of a systemic drug application, extracted thickness values for each cortical area were averaged across hemispheres. This procedure was additionally justified as cortical thickness in all ROIs was significantly correlated between the right and left hemisphere (Methods S5). Thickness measures within the ROIs were z-transformed on the basis of means and standard deviations of the control group (*n* = 38) for better comparisons between the ROIs.

### Statistical analysis

Demographic and drug use data were analyzed with Pearson’s *χ*^2^ tests, Students *t* tests, and analyses of variance (ANOVA), where appropriate. Group differences analyses in cognitive performance and cortical thickness were conducted by analyses of covariance (ANCOVA), followed by Sidak-corrected post hoc comparisons. In accordance with our previous study^15^, age and verbal IQ were introduced as covariates. Because ADHD has been linked to cognitive functioning in CU^15,28^, and to alterations in brain structure^36,37^, all ANCOVAs were additionally adjusted for the ADHD-SR score^24^. Given that lowLevCU and highLevCU 1) paid similar average prices for 1 g cocaine (Table 1, Table S3) and 2) reported comparable socioeconomic background in both studies (Table S4), socioeconomic status was not considered as a covariate. In the ANCOVAs that focused on the cocaine group comparison (lowLevCU vs. highLevCU), we introduced two further covariates: abstinence duration (as lowLevCU and highLevCU differed in self-reported days since last use, see Table 1) and cumulative cocaine dose because of the increased risk of cognitive impairment by ascending lifetime use of cocaine^15^. An additional cortical thickness analysis including duration of cocaine intake was calculated to control for differences between the two CU groups in Study 2 (Table S3). For correlation analyses the drug use parameters were log-transformed because they deviated from the normal distribution (Shapiro–Wilk *W* < 0.001). All confirmatory statistical comparisons were carried out on a significance level of *p* < 0.05.

**Table 1:** Demographic data and drug use pattern Study 1

	Controls (*n* = 78)	LowLevCU (*n* = 26)	HighLevCU (*n* = 49)	Value^a^	df, df_err_	*p*
Age (y)	30.2 (8.9)	33.0 (9.5)	31.5 (9.1)	*F* = 1.03	2.150	0.36
Sex (f/m)	23/55	7/19	11/38	*x*^2^ = 0.76	2	0.68
Verbal IQ (MWT-B)^b^	105.4 (9.2)	101.4 (8.7)	102.2 (10.7)	*F* = 2.50	2.150	0.09
Education (y)	10.7 (1.7)	9.8 (1.3)*	9.8 (1.7)**	*F* = 6.18	2.150	**0.003**
Smoking (y/n)^c^	57/21	23/3	39/10	*x*^2^ = 2.81	2	0.25
BDI score^d^	4.4 (4.4)	8.4 (6.1)*	9.6 (8.2)***	*F* = 12.25	2.150	**<** **0**.**001**
ADHD-SR score^e^	7.6 (4.7)	11.2 (6.3)	15.9 (9.1)***°	*F* = 23.78	2.150	**<** **0.001**
Cocaine						
Times per week^g^	—	2.0 (2.2)	1.8 (1.9)	*T* = 0.50	73	0.62
g per week^g^	—	3.8 (6.2)	3.3 (6.4)	*T* = 0.34	73	0.74
Years of use	—	7.7 (6.8)	8.6 (5.4)	*T* = −0.63	73	0.53
Maximum dose (g/day)	—	6.5 (6.7)	5.8 (6.2)	*T* = 0.48	73	0.63
Cumulative dose (g)	—	4130 (8272)	2658 (6689)	*T* = 0.83	73	0.41
Last consumption (days)^h^	—	29.4 (37.0)	13.3 (15.9)	*T* = 2.12	73	**0.04**
Urine toxicology (neg/pos)^i^	78/0	21/5	33/16	*x*^2^ = 1.52	1	0.22
Average price paid for 1 g (CHF)^j^ 1 g (CHF)^j^	—	97.5 (19.6)	87.5 (21.5)	*T* = 1.95	73	0.06
Hair analysis						
Cocaine pg/mg	—	10,261 (20,667)	12,993 (24,031)	*T* = −0.49	73	0.62
Benzoylecgonine pg/mg	—	2853 (6901)	2550 (4365)	*T* = 0.23	73	0.82
Norcocaine pg/mg	—	292 (655)	312 (484)	*T* = −0.15	73	0.88
Levamisole pg/mg	—	967 (1745)	6931 (11,737)	*T* = −3.48	73	**0.001**
Levamisole–cocaine ratio	—	0.12 (0.1)	0.64 (0.3)	*T* = −10.07	73	**<0.001**
Alcohol						
Pure ethanol g per week^g^	109.6 (121.9)	185.2 (281)	192.2 (204.5)*	*F* = 3.61	2.150	**0.03**
Years of use	12.6 (9.0)	11.7 (7.9)	13.3 (7.2)	*F* = 0.34	2.150	0.71
Nicotine						
Cigarettes per day^g^	8.8 (9.6)	16.7 (13.1)**	13.5 (10.3)*	*F* = 6.68	2.150	**0.002**
Years of use	8.4 (8.7)	13.6 (9.6)*	12.9 (8.5)*	*F* = 5.57	2.150	**0.005**
Cannabis						
g per week^g^	0.4 (0.9)	1.5 (4.0)	0.7 (1.7)	*F* = 2.81	2.150	0.06
Years of use	4.3 (5.7)	7.4 (9.2)	9.6 (7.7)***	*F* = 8.61	2.150	**<0.001**
Cumulative dose (g)	665 (3182)	3289 (7433)*	1823 (2886)	*F* = 4.18	2.150	**0.02**
Last consumption (days)^h^	41 (57);*n* *=* 34	31 (43);n = 14	25 (31);n = 34	*F* = 1.08	2.79	0.34
Urine toxicology (neg/pos)^i^	68/10	18/8	35/14	*x*^2^ = 6.35	2	**0.04**
Amphetamine						
g per week^g^	0.0 (0.0)	0.0 (0.1)	0.1 (0.2)*	*F* = 4.15	2.150	**0.02**
Years of use	0.0 (0.0)	1.1 (3.1)	1.5 (2.9)***	*F* = 8.23	2.150	**<0.001**
Cumulative dose (g)	0.0 (0.1)	6 (23.7)	28.4 (66.8)***	*F* = 8.14	2.150	**<0.001**
Last consumption (days)^h^	122 (0) *n* = 1	97 (71) *n* = 5	59 (54) *n* = 16	*F* = 1.23	2.19	0.31
Hair analysis pg/mg	1 (7)	24 (69)	118 (313)**	*F* = 6.57	2.150	**0.002**
MDMA						
Tablets per week^g^	0.0 (0.0)	0.0 (0.0)	0.1 (0.2)***°	*F* = 7.93	2.150	**<0.001**
Years of use	0.3 (1.7)	1.3 (2.4)	3 (4.5)***	*F* = 11.83	2.150	**<0.001**
Cumulative dose (tablets)	0.9 (3.2)	69.9 (154.3)*	54.1 (168.4)*	*F* = 5.21	2.150	**0.007**
Last consumption (days)^h^	5 (0) *n* = 1	92 (0) *n* = 1	71 (87) *n* = 17	*F* = 0.31	2.16	0.741
Hair analysis pg/mg	4 (23)	177 (337)	831 (1902)***°	*F* = 8.95	2.150	**<0.001**
Hallucinogens						
Cumulative dose (times)	0.7 (1.8)	9.7 (22.2)**	6.8 (10.5)**	*F* = 8.76	2.150	**<0.001**

Means and standard deviations. Significant *p* values are shown in bold

^a^ANOVA (all groups; significant Sidak post hoc test vs. control group: *p < 0.05; **p < 0.01; ***p < 0.001; vs. lowLevCU: °*p* < 0.05; °°*p* < 0.01); *x*² test (all groups/cocaine users only) for frequency data; Independent t-test (cocaine users only)

^b^Verbal IQ was assessed by the Mehrfachwahl–Wortschatz–Intelligenztest^25^

^c^Smoking habits were assessed by the Fagerstroem Test of Nicotine Dependence^63^

^d^*BDI* Beck Depression Inventory^64^

^e^*ADHD-SR* ADHD self-rating scale^24^

^f^Craving for cocaine was assessed by the Brief-CCQ^65^

^g^Average use during the last 6 months

^h^Last consumption is averaged only for persons who used the drug in the last 6 months. In this case, sample size (*n*) is shown

^i^Cut-off values for cocaine = 150 ng/ml and for Tetrahydrocannabinol 50 ng/ml^66^

^j^Price for 1 g cocaine in Swiss Francs paid by cocaine users (self-report). The quoted price is presumably below the real street price as some users paid reduced rates at intermediaries. Moreover, individuals who got the cocaine for free (e.g., as a gift) were excluded (*n* = 1 lowLevCU and *n* = 1 highLevCU)

## Results

### Study 1

#### Demographic characteristics and drug use

As intended by the matching procedure, the three groups did not differ regarding age, verbal IQ, sex distribution, and smoking status (Table 1). Additionally, there were no differences regarding the average price paid for 1 g of cocaine (Table 1) and socioeconomic status between both groups (Table S4). However, both CU groups had significantly fewer years of education and higher BDI scores than controls but did not differ from each other. Moreover, highLevCU displayed significantly higher ADHD-SR scores than lowLevCU. As a consequence of the group classification, the two CU groups differed strongly in their absolute levamisole concentrations and levamisole-related LCR but displayed similar values in any other cocaine-related hair toxicology or self-reported cocaine use parameter (with exception of abstinence duration). Additionally, hair samples and cumulative doses revealed a clear dominance of cocaine compared with other illegal drugs, as intended by the inclusion and exclusion criteria.

#### Neurocognitive measures

**Fig. 2:**
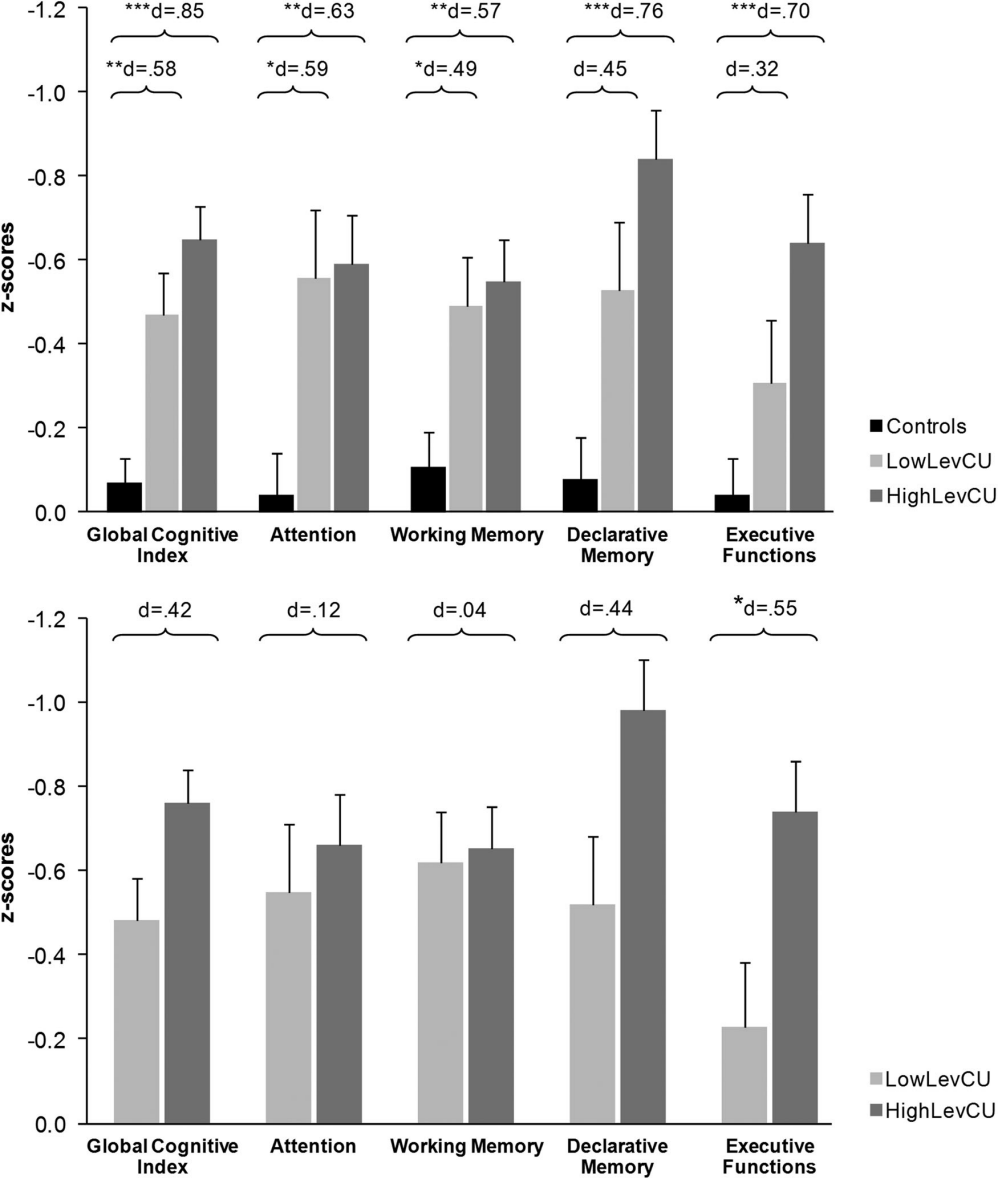
Mean z-scores and standard errors for the global cognitive index (GCI) and the four cognitive domains. **a** All values corrected for age, verbal IQ, and ADHD (based on all three groups). Sidak post hoc tests: *p < 0.05; **p < 0.01; ***p < 0.001. Cohen’s *d* vs. controls. **b** Cocaine user group values corrected for age, verbal IQ, ADHD, abstinence duration, and cumulative cocaine dose (based on cocaine user groups). Sidak post hoc tests: *p < 0.05. Cohen’s d lowLevCU vs. highLevCU

As shown before in this sample^15^, controls and CU (lowLevCU + highLevCU) differed significantly in the GCI and all four domains (F(1148) = 10.64–28.34, *p* ≤ 0.001) (Table S5). Three-group ANCOVAs (controls vs. lowLevCU vs. highLevCU) for the GCI (F(2147) = 15.26, *p* < 0.001) and across all four cognitive domains (F(2147) = 6.70–10.45, *p* = 0.002–0.0001) showed significant group effects (Fig. 2a, Table S6). Linear trends across groups were shown for all comparisons (*p* < 0.01–0.001), suggesting not only a cocaine but also a levamisole effect on cognitive functioning. The post hoc pairwise comparisons showed that lowLevCU differed from controls in the GCI, attention, and working memory domain, while highLevCU differed from controls in all cognitive domains (Fig. 2a). In general, effect sizes were considerably higher for highLevCU (*d* = 0.57–0.80) compared to lowLevCU (*d* = 0.32–0.59). Subsequently, to adjust for even subtle differences in cocaine use intensity, both CU groups were compared using ANCOVAs in which abstinence duration und cumulative lifetime dose of cocaine were additionally included. Here, highLevCU showed a stronger impairment of executive functions with a medium effect size compared to lowLevCU (*F*(1,68) = 5.02, *p* < 0.05, *d* = 0.55). Additionally, the GCI (*F*(1,68) = 3.21, *p* = 0.08, 0.42) and declarative memory (*F*(1,68) = 3.21, *p* = 0.08, *d* = 0.44) showed statistical trends towards significance with approximately medium effect sizes (Fig. 2b). The impact on executive function was mainly driven by a worse performance in the IDE task and recall consistency (Table S7), indicating more pronounced impairments specifically in rule acquisition and reversal learning as well as in memory organization in highLevCU. An exploratory analysis of the IDE stages revealed that highLevCU made more errors specifically in the intradimensional set-shifting (pre-ED errors: (*F*(1,68) = 0.01, *p* < 0.05, *d* = 0.64) but not in the extradimensional set-shifting (ED errors: *F*(1,68) = 6.02, *p* = 0.94, *d* = 0.02; Figure S3). Notably, in a combined CU group, the executive function performance correlated negatively with the log-transformed levamisole values in hair samples (*r* = −0.23, *p* < 0.05, one-tailed; Figure S4).

### Study 2

#### Demographic characteristics and levamisole analysis

Again, the three groups did not differ regarding education, sex distribution, smoking status, average price paid for 1 g of cocaine (Table S3), and socioeconomic status (Table S4). As in Study 1, the two CU groups showed higher BDI and ADHD-SR scores than healthy controls. Moreover, the lowLevCU had a significant lower verbal IQ than the highLevCU group and the controls. Hair toxicology measures between the two CU groups did only differ for the measured levamisole concentration as well as the levamisole-related LCR.

#### Thickness measures

**Fig. 3:**
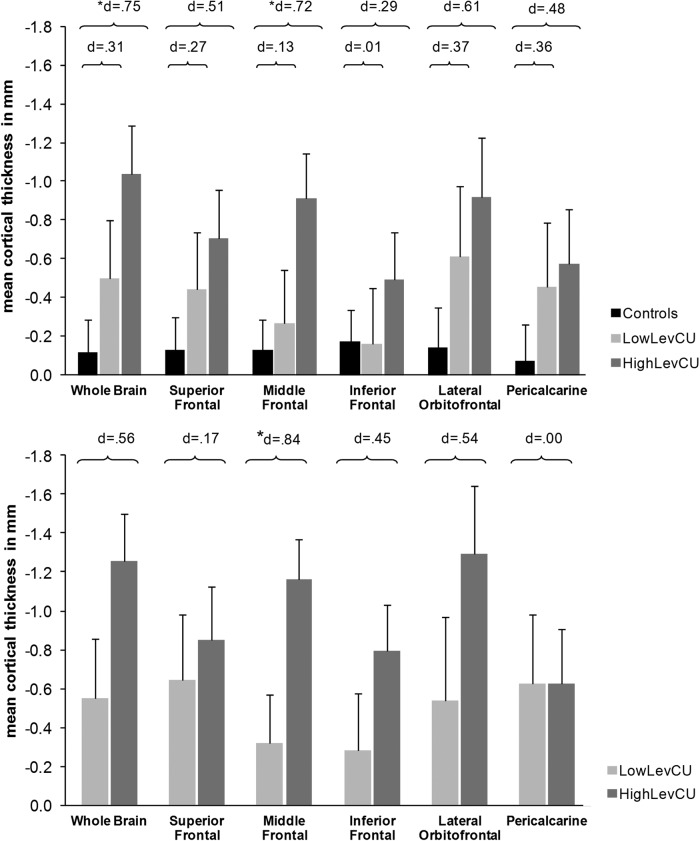
Mean cortical thickness (in mm) and standard errors for the whole brain and five regions of interest. **a** All values corrected for age, verbal IQ, and ADHD (based on all three groups). Sidak post hoc tests: *p < 0.05. Cohen’s *d* vs. controls. **b** Cocaine user group values corrected for age, verbal IQ, ADHD, abstinence duration, and cumulative cocaine dose (based on cocaine user groups). Sidak post hoc tests: *p < 0.05. Cohen’s d lowLevCU vs. highLevCU

Three-group comparisons revealed significant group effects on cortical thickness for the whole brain ROI (*F*(2,61) = 3.90, *p* < 0.05), and the MFG (*F*(2,61) = 3.61, *p* < 0.05; Fig. 3a, Table S1). Both measures showed significant linear trends across groups (*p* < 0.05) and post hoc pairwise comparisons indicated that cortical thickness was significantly decreased in highLevCU compared to controls. As in Study 1 two additional cocaine-related covariates were added for the two-group ANCOVAs (lowLevCU vs. highLevCU): abstinence duration and cumulative lifetime dose (Fig. 3b, Table S2). A significant group difference was found for the MFG showing a strong effect size (*F*(1,22) = 5.65, *p* < 0.05, *d* = 0.84). This effect remained significant, when alcohol (pure ethanol in g per week) was considered as an additional covariate (*F*(1,21) = 5.16, *p* < 0.05, *d* = 0.80) of potential impact on cortical thickness. ANCOVAs for whole brain, IFG, and lOFG—albeit not statistically significant—showed medium effect sizes (*F*(1,22) = 1.52–2.74, *p* = 0.23–0.11, *d* = 0.45–0.56). A small effect was applicable for the SFG (*F*(1,22) = 0.18, *p* = 0.67, *d* = 0.17). By contrast, no effect was found for the pericalcarine gyrus as expected (*F*(1,22) = 0.00, *p* = 0.99, *d* = 0.00). Importantly, MFG thickness was negatively correlated with the log-transformed levamisole hair concentration (*r* = −0.32, *p* < 0.05, one-tailed; Figure S5).

## Discussion

The aim of the present studies was to examine whether the worldwide highly prevalent cocaine adulterant levamisole is associated with higher risks for cognitive impairment and structural brain alterations in chronic CU with recent levamisole exposure. We first demonstrated that highLevCU showed significantly worse executive functions (Cohen’s *d* = 0.55) compared to individuals with equivalent cocaine use intensity but lower levamisole hair concentrations. Although not significant, similar patterns with approximately medium effect sizes were also found for the global cognition score (*d* = 0.42) and declarative memory performance (*d* = 0.44), but not for attention (*d* = 0.12) and working memory (*d* = 0.04). Notably, compared to stimulant-naive healthy controls, significant cognitive deficits were still present in CU with low levamisole exposure. Based on these initial findings, we subsequently performed a second study employing structural MRI analyses. In line with the results from the cognitive study, we found significantly reduced cortical thickness in the MFG of CU with high levamisole hair concentrations (*d* = 0.84). Moreover, even though not statistically significant-related effects were shown for the whole brain (*d* = 0.56), IFG (*d* = 0.45), and lOFG (*d* = 0.54), while in an occipital control region no levamisole effect was observable (*d* = 0.00).

In sum, these findings confirm our previous proposition^15,16^ that cocaine use is linked with broad cognitive impairments in the present sample. However, also the adulterant levamisole seems to be related to these impairments, most strongly in the executive functions but also in declarative memory and global cognitive functions. Moreover, levamisole-associated reductions of cortical thickness were also found in lateral frontal brain areas, indicating possible neuroanatomical underpinnings of executive function deficits found in highLevCU. In line with an early animal study^12^, these results suggest that levamisole is linked to neurotoxic effects also in humans with regular use of levamisole-contaminated cocaine. Importantly, because highLevCU and lowLevCU did not differ in their socioeconomic background and paid comparable prices for their street cocaine, low income is likely not an alternative explanation for the cognitive and cortical alterations found in cocaine users with high levamisole exposure.

Previous studies consistently showed strong deficits of CU in attention and working memory, whereas the heterogeneous concept of executive functions was usually less affected^15,38–40^. Here, we also found clear cocaine but no pronounced levamisole effects in the domains of attention and working memory but a significant levamisole effect on executive functions. Thus, one might speculate that at least some of the reported discrepant findings in the newer literature regarding executive function impairments^41^ might be explained by differences in recent levamisole exposure. As levamisole was proposed to be metabolized into the amphetamine-like stimulant aminorex and other metabolites^1,6,7^, and previous reports showed pronounced executive function decrements in chronic amphetamine users^42,43^, the present effect might not be linked to levamisole alone but also to its metabolic products.

The indicated levamisole effect on the executive function domain was mainly driven by low performance in an attentional set-shifting/reversal learning task (IED) and worse recall consistency in a verbal learning task (RAVLT), while the strategy score of a spatial working memory task (SWM) was less affected. This supports the assumption that levamisole might have little effect on working memory processes per se but impacts cognitive flexibility and memory organization. Remarkably, these specific cognitive impairments are well in line with the found structural alterations in the MFG, given that (1) the MFG is prominently involved in attentional set-shifting and reversal learning^44,45^ and (2) patients with focal frontal lesions have difficulties in memory organization such as recall consistency^18,19^. Moreover, frontal lobe atrophy has been shown as the most consistent predictors for recall consistency in patients with multiple sclerosis^46^. Finally, age-related changes presumably of the prefrontal cortex (including predominantly the MFG)^47,48^ as well as excitotoxic prefrontal lesions^49^ are associated with impairments in set-shifting in monkey models.

To date, the exact neurobiological substrates behind the cocaine-related cognitive alterations are still not fully understood^50^. Cocaine is an unspecific monoamine reuptake inhibitor with high affinity for dopamine, serotonin, and norepinephrine transporters (DAT, SERT, and NET)^51^. Thus, cognitive deficits most likely depend on adaptions involving regions with high concentrations of monoamine responsive cells such as the prefrontal cortex^52^. Also the exact neurobiological effects of levamisole remain unclear. Recent research suggested that levamisole has only minor effects on monoamine transporter^6^. Yet, the metabolite aminorex, has a similar affinity to NET and DAT as cocaine, while showing less binding to the SERT^6^. However, it is not fully clear if aminorex is able to augment cocaine effects in humans in general, but due to its longer half-life it might at least prolong the stimulant effects of cocaine^6,53^. Interestingly, specific impairments in attentional set-shifting were reported for noradrenergic but not cholinergic deafferentation of the medial prefrontal cortex—the homolog of the primate dorsolateral prefrontal cortex in rats^54^. Given that we previously proposed that CU might show neuroplastic adaptations in the noradrenaline system^55,56^ one could speculate that not only cocaine but specifically a cocaine–aminorex combination can disrupt the noradrenaline transmission. Moreover, medically prescribed levamisole intake is supposed to cause multifocal inflammatory leukoencephalopathy^57,58^, a disease associated with white matter lesions. Thus, executive function impairments might be mainly explained by levamisole (or its metabolites) as white matter lesions are associated with cognitive dysfunctions in general^59^ and executive function deficits in particular^60^. Importantly, executive function deficits are also strongly linked to gray matter alterations in the prefrontal cortex^61^. Thus, executive function deficits in highLevCU are likely explained by neuroanatomical alterations of the prefrontal cortex beyond the cortical abnormalities linked to cocaine consumption per se^42^.

A limitation of this study is that the objective hair toxicology parameters covered only the last 3 to 6 months. Consequently, the group classification based on the LCR reflected a recent but not necessarily a long-term levamisole exposure. Moreover, we did not apply a neuropsychological test battery in Study 2 at the time of structural imaging and, thus, were not able to directly correlate cognitive performance with cortical thickness scores. Finally, the applied cross-sectional case–control study design makes it impossible to determine the causal relationship between levamisole and neurocognitive and imaging measures.

In conclusion, CU with high levamisole exposure showed significantly worse executive functioning than CU with comparable cocaine use severity but low levamisole contamination. Moreover, high levamisole exposure was associated with lower cortical thickness, primarily for the MFG but also—even though not statistically significant—in additional frontal regions and on a whole brain level. Altogether, our results indicate that exposure to high doses of levamisole during the last months (covered by the hair analyses) goes along with pronounced neurocognitive and cortical alterations in CU, strongly indicating a possible neurotoxic effect of levamisole in humans. Consequently, CU should be better informed about the consequences of levamisole-adulterated cocaine and drug policy makers should consider prevention and harm reduction programs, which lead to a reduction of levamisole contamination of street cocaine such as drug-checking services for users^62^.

### Disclaimer

M.V., S.H., and B.B.Q. have full access to all of the data in the study and take responsibility for the integrity of the data and the accuracy of the data analysis.

## Electronic supplementary material

Supplemental Information

## References

[1] Brunt TM, van den Berg J, Pennings E, Venhuis B (2017). Adverse effects of levamisole in cocaine users: a review and risk assessment. Arch. Toxicol..

[2] European Monitoring Centre for Drugs and Drug Addiction. (2010). Annual Report 2010: The State of The Drug Problem in Europe.

[3] Eiden C, Diot C, Mathieu O, Mallaret M, Peyriere H (2014). Levamisole-adulterated cocaine: what about in European countries?. J. Psychoact. Drugs.

[4] The Drug Enforcement Administration. National Drug Threat Assessment 2017, October 2017 edn. (U.S. Drug Enforcement Administration, Springfield, VA, 2017).

[5] United Nations Office on Drugs and Crime. (2011). World Drug Report 2011.

[6] Hofmaier T (2014). Aminorex, a metabolite of the cocaine adulterant levamisole, exerts amphetamine like actions at monoamine transporters. Neurochem. Int..

[7] Kudlacek O (2017). Cocaine adulteration. J. Chem. Neuroanat..

[8] Zimmermann ZJ, Gauvin DV, Poling A (2018). Discriminative stimulus effects of cocaine-levamisole combinations in Sprague-Dawley rats. J. Psychopharmacol..

[9] Larocque A, Hoffman RS (2012). Levamisole in cocaine: unexpected news from an old acquaintance. Clin. Toxicol..

[10] Vitt JR, Brown EG, Chow DS, Josephson SA (2017). Confirmed case of levamisole-associated multifocal inflammatory leukoencephalopathy in a cocaine user. J. Neuroimmunol..

[11] Lee KC, Ladizinski B, Federman DG (2012). Complications associated with use of levamisole-contaminated cocaine: an emerging public health challenge. Mayo Clin. Proc..

[12] Vandevelde M, Boring JG, Hoff EJ, Gingerich DA (1978). The effect of levamisole on the canine central nervous system. J. Neuropathol. Exp. Neurol..

[13] Hook CC (1992). Multifocal inflammatory leukoencephalopathy with 5-fluorouracil and levamisole. Ann. Neurol..

[14] Lynch KL, Dominy SS, Graf J, Kral AH (2011). Detection of levamisole exposure in cocaine users by liquid chromatography-tandem mass spectrometry. J. Anal. Toxicol..

[15] Vonmoos M (2013). Cognitive dysfunctions in recreational and dependent cocaine users: role of attention-deficit hyperactivity disorder, craving and early age at onset. Br. J. Psychiatry.

[16] Vonmoos M (2014). Cognitive impairment in cocaine users is drug-induced but partially reversible: evidence from a longitudinal study. Neuropsychopharmacology.

[17] Hulka LM (2015). Changes in cocaine consumption are associated with fluctuations in self-reported impulsivity and gambling decision-making. Psychol. Med..

[18] Alexander MP, Stuss DT, Fansabedian N (2003). California verbal learning test: performance by patients with focal frontal and non-frontal lesions. Brain.

[19] Stuss DT (1994). Organizational strategies with unilateral or bilateral frontal lobe injury in word learning tasks. Neuropsychology.

[20] Robbins TW (1998). A study of performance on tests from the CANTAB battery sensitive to frontal lobe dysfunction in a large sample of normal volunteers: implications for theories of executive functioning and cognitive aging. Cambridge Neuropsychological Test Automated Battery. J. Int. Neuropsychol. Soc..

[21] Dias R, Robbins TW, Roberts AC (1996). Dissociation in prefrontal cortex of affective and attentional shifts. Nature.

[22] American Psychological Association. (1994). Diagnostic and Statistical Manual of Mental Disorders: DSM-IV.

[23] Quednow BB, Kuhn KU, Hoenig K, Maier W, Wagner M (2004). Prepulse inhibition and habituation of acoustic startle response in male MDMA (‘ecstasy’) users, cannabis users, and healthy controls. Neuropsychopharmacology.

[24] Rosler M (2004). [Tools for the diagnosis of attention-deficit/hyperactivity disorder in adults. Self-rating behaviour questionnaire and diagnostic checklist]. Nervenarzt.

[25] Lehrl S (2005). Mehrfachwahl-Wortschatz-Intelligenztest MWT-B. Fünfte Auflage.

[26] Wechsler D. A. *Wechsler Memory Scale. Technical Manual*, 3rd edn (Psychological Cooperation, San Antonio, TX, 1997).

[27] Helmstaedter C, Lendt M, Lux S (2001). Verbaler Lern und Merkfähigkeitstest (Verbal learning and memory test).

[28] Wunderli MD (2016). Cognitive and emotional impairments in adults with attention-deficit/hyperactivity disorder and cocaine use. Drug Alcohol Depend..

[29] Dale AM, Fischl B, Sereno MI (1999). Cortical surface-based analysis. I. Segmentation and surface reconstruction. Neuroimage.

[30] Fischl B, Sereno MI, Dale AM (1999). Cortical surface-based analysis. II: Inflation, flattening, and a surface-based coordinate system. Neuroimage.

[31] Reuter M, Rosas HD, Fischl B (2010). Highly accurate inverse consistent registration: a robust approach. Neuroimage.

[32] Desikan RS (2006). An automated labeling system for subdividing the human cerebral cortex on MRI scans into gyral based regions of interest. Neuroimage.

[33] Cristofori I (2015). White and gray matter contributions to executive function recovery after traumatic brain injury. Neurology.

[34] Lewis DA (2001). Dopamine transporter immunoreactivity in monkey cerebral cortex: regional, laminar, and ultrastructural localization. J. Comp. Neurol..

[35] Fischman AJ (2001). [(11)C, (127)I] Altropane: a highly selective ligand for PET imaging of dopamine transporter sites. Synapse.

[36] Onnink AM (2014). Brain alterations in adult ADHD: effects of gender, treatment and comorbid depression. Eur. Neuropsychopharmacol..

[37] Almeida LG (2010). Reduced right frontal cortical thickness in children, adolescents and adults with ADHD and its correlation to clinical variables: a cross-sectional study. J. Psychiatr. Res..

[38] Jovanovski D, Erb S, Zakzanis KK (2005). Neurocognitive deficits in cocaine users: a quantitative review of the evidence. J. Clin. Exp. Neuropsychol..

[39] Potvin S, Stavro K, Rizkallah E, Pelletier J (2014). Cocaine and cognition: a systematic quantitative review. J. Addict. Med.

[40] Spronk DB, van Wel JH, Ramaekers JG, Verkes RJ (2013). Characterizing the cognitive effects of cocaine: a comprehensive review. Neurosci. Biobehav. Rev..

[41] Vonmoos M., Quednow B.B. (2017). Cognitive Dysfunctions in Chronic Cocaine Users. The Neuroscience of Cocaine.

[42] Ersche KD, Williams GB, Robbins TW, Bullmore ET (2013). Meta-analysis of structural brain abnormalities associated with stimulant drug dependence and neuroimaging of addiction vulnerability and resilience. Curr. Opin. Neurobiol..

[43] van Holst RJ, Schilt T (2011). Drug-related decrease in neuropsychological functions of abstinent drug users. Curr. Drug Abus. Rev..

[44] Oh A, Vidal J, Taylor MJ, Pang EW (2014). Neuromagnetic correlates of intra- and extra-dimensional set-shifting. Brain Cogn..

[45] Rogers RD, Andrews TC, Grasby PM, Brooks DJ, Robbins TW (2000). Contrasting cortical and subcortical activations produced by attentional-set shifting and reversal learning in humans. J. Cogn. Neurosci..

[46] Benedict RH (2005). Regional lobar atrophy predicts memory impairment in multiple sclerosis. Am. J. Neuroradiol..

[47] Moore TL (2005). Cognitive impairment in aged rhesus monkeys associated with monoamine receptors in the prefrontal cortex. Behav. Brain. Res..

[48] Moore TL, Killiany RJ, Herndon JG, Rosene DL, Moss MB (2006). Executive system dysfunction occurs as early as middle-age in the rhesus monkey. Neurobiol. Aging.

[49] Dias R, Robbins TW, Roberts AC (1996). Primate analogue of the Wisconsin Card Sorting Test: effects of excitotoxic lesions of the prefrontal cortex in the marmoset. Behav. Neurosci..

[50] Cadet, J. L. & Bisagno, V. Neuropsychological consequences of chronic drug use: relevance to treatment approaches. *Front. Psychiatry* **6**, 189 (2015). 10.3389/fpsyt.2015.00189 PMC4713863 26834649

[51] Ritz MC, Cone EJ, Kuhar MJ (1990). Cocaine inhibition of ligand binding at dopamine, norepinephrine and serotonin transporters: a structure-activity study. Life Sci..

[52] Nestler EJ (2005). The neurobiology of cocaine addiction. Sci. Pract. Perspect..

[53] Hess C, Ritke N, Broecker S, Madea B, Musshoff F (2013). Metabolism of levamisole and kinetics of levamisole and aminorex in urine by means of LC-QTOF-HRMS and LC-QqQ-MS. Anal. Bioanal. Chem..

[54] McGaughy J, Ross RS, Eichenbaum H (2008). Noradrenergic, but not cholinergic, deafferentation of prefrontal cortex impairs attentional set-shifting. Neuroscience.

[55] Preller KH (2013). Increased sensorimotor gating in recreational and dependent cocaine users is modulated by craving and attention-deficit/hyperactivity disorder symptoms. Biol. Psychiatry.

[56] Havranek MM (2017). alpha2A -Adrenergic receptor polymorphisms and mRNA expression levels are associated with delay discounting in cocaine users. Addict. Biol..

[57] Wu VC (2006). Levamisole-induced multifocal inflammatory leukoencephalopathy: clinical characteristics, outcome, and impact of treatment in 31 patients. Medicine.

[58] Xu N (2009). Clinical and MRI characteristics of levamisole-induced leukoencephalopathy in 16 patients. J. Neuroimaging.

[59] Filley CM, Fields RD (2016). White matter and cognition: making the connection. J. Neurophysiol..

[60] Desmond DW (2002). Cognition and white matter lesions. Cerebrovasc. Dis..

[61] Yuan P, Raz N (2014). Prefrontal cortex and executive functions in healthy adults: a meta-analysis of structural neuroimaging studies. Neurosci. Biobehav. Rev..

[62] Hungerbuehler I, Buecheli A, Schaub M (2011). Drug checking: a prevention measure for a heterogeneous group with high consumption frequency and polydrug use - evaluation of zurich’s drug checking services. Harm. Reduct. J..

[63] Heatherton TF, Kozlowski LT, Frecker RC, Fagerstrom KO (1991). The Fagerstrom test for nicotine dependence: a revision of the Fagerstrom Tolerance Questionnaire. Br. J. Addict..

[64] Hautzinger M, Bailer M, Worall H, Keller F (1994). Beck Depression Inventory. Test Manual.

[65] Sussner BD (2006). The validity and reliability of a brief measure of cocaine craving. Drug Alcohol. Depend..

[66] Substance Abuse and Mental Health Services Administration. (2008). Mandatory Guidelines for federal workplace drug testing programs. Fed. Regist..

