# Supplementary material for: Cognitive and neuroanatomical impairments associated with chronic exposure to levamisole-contaminated cocaine
Source: Transl Psychiatry. 2018 Oct 27;8:235. doi: 10.1038/s41398-018-0279-3 (PMC6204136; doi:10.1038/s41398-018-0279-3)
Supplement: Supplementary file 1 — Supplemental Information [file 41398_2018_279_MOESM1_ESM.docx]

**Supplemental Information**

**Cognitive and neuroanatomical impairments associated with chronic exposure to levamisole-contaminated cocaine**

Matthias Vonmoos^1^*, PhD; Sarah Hirsiger^1^, PhD; Katrin H. Preller^1^, PhD; Lea M. Hulka^1^, PhD;
Daniel Allemann^2^, PhD; Marcus Herdener^3^, MD; Markus R. Baumgartner^4^, PhD; Boris B. Quednow^1^*, PhD

## Methods S1. Recruitment and selection Study 1.

The recruitment focused on the greater area of Zurich and lasted from January 2010 until October 2012. Participants were recruited via advertisements in local newspapers, online media, drug prevention and treatment centers, psychiatric hospitals, and by word of mouth. After initial standardized telephone interviews, 250 subjects (108 psychostimulant-naïve controls, 142 CU) were considered to be eligible for inclusion in the cross-sectional sample. All subjects were aged between 18 and 60 years and had sufficient German language skills. Seventy-one participants were excluded because hair analyses revealed illegal drug use not declared in the interviews (e.g., opioids, excessive MDMA use) or due to a lack of cocaine use. Twelve controls were excluded due to matching reasons (age, verbal IQ, and smoking) between groups.

Furthermore, the data of four participants could not be analyzed because of technical problems during the test session and ten participants provided no or not enough hair to be toxicologically analyzed. This lead to a final sample of 75 CU and 78 healthy controls.

***Methods S2.*** Urine and hair toxicology analyses.

Urine toxicology analyses comprised the compounds/substances: tetrahydrocannabinol, cocaine, amphetamines, benzodiazepines, opioids, and methadone and were assessed by a semi-quantitative enzyme multiplied immunoassay method using a Dimension RXL Max (Siemens, Erlangen, Germany).

To characterize drug use and levamisole exposure over the last months objectively, hair samples were collected and analyzed with liquid chromatography-tandem mass spectrometry (LC-MS/MS). If participants’ hair was long enough, one sample of six cm hair (from the scalp) was taken from the occiput and subsequently divided into two subsamples of three cm length. The following compounds were assessed: levamisole, cocaine, benzoylecgonine, ethylcocaine, norcocaine, amphetamine, methamphetamine, MDMA, MDEA, MDA, ephedrine, morphine, codeine, dihydrocodeine, methadone EDDP (primary methadone metabolite), oxycodone, tramadol, and methylphenidate.

For our routine protocol a three step washing procedure with water (2min shaking, 15ml), acetone (2min, 10ml) and finally hexane (2min, 10ml) of hair was performed. Then the hair samples were dried at ambient temperatures, cut into small snippets and extracted in two steps, first with methanol (5ml, 16h, ultrasonication) and a second step with 3ml MeOH acidified with 50µl hydrochloric acid 33% (3h, ultrasonication). The extracts were dried and the residue reconstituted with 50µl MeOH and 500µl 0.2mM ammonium formate (analytical grade) in water. As internal standards deuterated standards of the following compounds were used, added as mixture of the following compounds: cocaine-d3, benzoylecgonine-d3, ethylcocaine-d3, morphine-d3, MAM-d3, codeine-d3, dihydrocodeine-d3, amphetamine-d6, methamphetamine-d9, MDMA-d5. MDEA-d6, MDA-d5, methadone-d9, EDDP-d3, methylphenidate-d9, tramadol-d3, oxycodone-d3, and ephedrine-d3. All deuterated standards were from ReseaChem (Burgdorf, Switzerland), the solvents for washing and extraction were of analysis grade and obtained from Merck (Darmstadt, Germany); LC-solvents were of HPLC grade and were obtained from Sigma Aldrich (Buchs, Switzerland).

The LC-MS/MS apparatus was an ABSciex QTrap 3200 (Analyst software Version 1.5, Turbo V ion source operated in the ESI mode, gas 1, nitrogen (50psi); gas 2, nitrogen (60psi); ion spray voltage, 3500V; ion source temperature, 450°C; curtain gas, nitrogen (20psi) collision gas, medium), with a Shimadzu Prominence LC-system (Shimadzu CBM 20 A controller, two Shimadzu LC 20 AD pumps including a degasser, a Shimadzu SIL 20 AC autosampler and a Shimadzu CTO 20 AC column oven, Shimadzu, Duisburg, Germany). Gradient elution was performed on a separation column (Synergi 4µ POLAR-RP 80A, 150x2.0 with a POLAR-RP 4x2.0 Security Guard Cartridge, (Phenomenex, Aschaffenburg, Germany). The mobile phase consisted of 1mM ammonium formate buffer adjusted to pH 3,5 with formic acid (eluent A) and acetonitrile containing 1mM ammonium formate and 1mM formic acid (eluent B). The analysis was performed in MRM mode with two transitions per analyte and one transition for each deuterated internal standard, respectively.

The above mentioned substances were measured in a first analytical step. In a second step, we analyzed levamisole by the same method. This additional levamisole measurement (definition of MRM measurement parameters and retention time) based on the internal standard of cocaine-d3 (calibration between reference measurement an 25‘000 pg/mg, 4 calibrators, linear, r^2^=.99).

## Methods S3. Recruitment and selection Study 2.

A subsample 17 individuals of Study 1 also participated in Study 2. The remaining 61 participants were re-invited from an additional cross-sectional study, which was conducted in the Zurich area between November 2015 and November 2016. As in Study 1, all participants were aged between 18 and 60 and had sufficient German language skills. Six participants had to be excluded despite reporting stable consume over 0.5g per month their hair concentration of cocaine did not reach the detectable level of 500 pg/mg. One CU provided no hair sample and one participant had to be excluded as the hair analysis revealed a clear polydrug use pattern. Unexpectedly, one control subject had to be excluded due to a positive urine sample for opioids, one due to cocaine traces in hair, and one due to regular intake of strong migraine medication. Leading to a final sample of 29 CU and 38 healthy controls.

## Methods S4. FreeSurfer details.

In summary, the processing pipeline included the following steps: motion correction, automated Talairach transformation, non-parametric non-uniform intensity normalization (N3), removal of non-brain tissue (skull stripping), and generation of individual cortical surface models. Once the surfaces were reconstructed, several anatomical parameters were extracted at each vertex of the tessellated surface. Cortical thickness was defined as the minimal distance between the white/gray matter boundary and the pial surface[^1^](#_ENREF_1) and was formerly validated by using manual segmentations[^2^](#_ENREF_2).

## Methods S5. ROI-correlations between the right and the left hemisphere.

Mean thickness: r=.97, p<.001; superior frontal gyrus: r=.84, p<.001; middle frontal gyrus: r=.76, p<.001; inferior frontal gyrus: r=.75, p<.001; lateral orbitofrontal gyrus: r=.55, p<.001; pericalcarine gyrus: r=.62, p<.001.

***Table S1.*** Cortical thickness measures Study 2. Three-group-comparison.

|  |  |  |  |  |  |  | Cohen's d | | |
| --- | --- | --- | --- | --- | --- | --- | --- | --- | --- |
| Mean cortical thickness measures | Controls | LowLevCU | HighLevCU | F | df, df_err_ | p | Controls  vs. LowLevCU | Controls  vs. HighLevCU | LowLevCU  vs. HighLevCU |
|  |  |  |  |  |  |  |  |  |  |
| *All participants included* | n=38 | n=12 | n=17 |  |  |  |  |  |  |
|  |  |  |  |  |  |  |  |  |  |
| Whole brain | 2.51 (0.02) | 2.48 (0.03) | 2.43 (0.02)* | 3.90 | 2,61 | **.03** | .31 | .75 | .44 |
| Superior frontal | 2.84 (0.02) | 2.79 (0.04) | 2.76 (0.03) | 1.603 | 2,61 | .21 | .27 | .51 | .24 |
| Middle frontal | 2.49 (0.02) | 2.47 (0.03) | 2.40 (0.03)* | 3.605 | 2,61 | **.03** | .13 | .72 | .60 |
| Inferior frontal | 2.64 (0.02) | 2.64 (0.04) | 2.59 (0.03) | 0.669 | 2,61 | .52 | .01 | .29 | .30 |
| Lateral orbitofrontal | 2.64 (0.02) | 2.59 (0.04) | 2.56 (0.03) | 1.958 | 2,61 | .15 | .37 | .61 | .24 |
| Pericalcarine | 1.57 (0.02) | 1.53 (0.03) | 1.52 (0.03) | 1.045 | 2,61 | .36 | .36 | .48 | .12 |
|  |  |  |  |  |  |  |  |  |  |
| *Without alcohol dependent participants* | n=38 | n=9 | n=14 |  |  |  |  |  |  |
|  |  |  |  |  |  |  |  |  |  |
| Whole brain | 2.51 (0.01) | 2.47 (0.03) | 2.44 (0.02)* | 3.214 | 2,55 | **.05** | .39 | .72 | .33 |
| Superior frontal | 2.84 (0.02) | 2.79 (0.04) | 2.77 (0.04) | 1.251 | 2,55 | .29 | .34 | .44 | .09 |
| Middle frontal | 2.49 (0.02) | 2.47 (0.03) | 2.41 (0.03) | 2.928 | 2,55 | *.06* | .14 | .70 | .56 |
| Inferior frontal | 2.64 (0.02) | 2.63 (0.05) | 2.60 (0.04) | 0.372 | 2,55 | .69 | .06 | .26 | .20 |
| Lateral orbitofrontal | 2.64 (0.02) | 2.59 (0.04) | 2.58 (0.03) | 1.351 | 2,55 | .28 | .40 | .50 | .10 |
| Pericalcarine | 1.57 (0.02) | 1.49 (0.04) | 1.53 (0.03) | 1.954 | 2,55 | .15 | .80 | .40 | .40 |
|  |  |  |  |  |  |  |  |  |  |
| *Without opioid using participants* | n=38 | n=12 | n=15 |  |  |  |  |  |  |
|  |  |  |  |  |  |  |  |  |  |
| Whole brain | 2.51 (0.02) | 2.47 (0.03) | 2.42 (0.03)* | 4.384 | 2,59 | **.02** | .34 | .83 | .49 |
| Superior frontal | 2.84 (0.02) | 2.79 (0.04) | 2.75 (0.04) | 1.738 | 2,59 | .19 | .29 | .55 | .26 |
| Middle frontal | 2.49 (0.02) | 2.47 (0.03) | 2.40 (0.03)* | 3.929 | 2,59 | **.03** | .17 | .80 | .63 |
| Inferior frontal | 2.64 (0.02) | 2.64 (0.04) | 2.58 (0.04) | 0.95 | 2,59 | .39 | .01 | .36 | .37 |
| Lateral orbitofrontal | 2.64 (0.02) | 2.59 (0.04) | 2.56 (0.04) | 1.7 | 2.59 | .19 | .34 | .60 | .26 |
| Pericalcarine | 1.56 (0.02) | 1.53 (0.03) | 1.52 (0.03) | 0.747 | 2,59 | .48 | .32 | .42 | .10 |
|  |  |  |  |  |  |  |  |  |  |

Estimated means (in mm) and standard errors. ANCOVA (all groups, corrected for age, verbal IQ, and ADHS-SR sum score). Significant p values are shown in bold. Significant Sidak post-hoc test vs. control group: *p<.05; **p<.01; ***p<.001.

***Table S2.*** Cortical thickness measures Study 2. Cocaine user group comparison.

|  | LowLevCU | HighLevCU | F | df, df_err_ | p | Cohen's d |
| --- | --- | --- | --- | --- | --- | --- |
|  |  |  |  |  |  |  |
| *All participants included* | n=12 | n=17 |  |  |  |  |
|  |  |  |  |  |  |  |
| Whole brain | 2.47 (0.03) | 2.41 (0.02) | 2.74 | 1,22 | .11 | .56 |
| Superior frontal | 2.76 (0.05) | 2.74 (0.04) | 0.18 | 1,22 | .67 | .17 |
| Middle frontal | 2.47 (0.03) | 2.38 (0.02) | 5.65 | 1,22 | **.03** | .84 |
| Inferior frontal | 2.62 (0.04) | 2.55 (0.03) | 1.52 | 1,22 | .23 | .45 |
| Lateral orbitofrontal | 2.60 (0.04) | 2.52 (0.04) | 1.55 | 1,22 | .23 | .54 |
| Pericalcarine | 1.51 (0.03) | 1.51 (0.03) | 0.00 | 1,22 | .99 | .00 |
|  |  |  |  |  |  |  |
| *Duration included as additional covariate* | n=12 | n=17 |  |  |  |  |
|  |  |  |  |  |  |  |
| Whole brain | 2.47 (0.03) | 2.40 (0.02) | 3.22 | 1,21 | .09 | .59 |
| Superior frontal | 2.77 (0.04) | 2.73 (0.03) | 0.35 | 1,21 | .56 | .22 |
| Middle frontal | 2.47 (0.03) | 2.37 (0.02) | 5.67 | 1,21 | **.03** | .86 |
| Inferior frontal | 2.63 (0.04) | 2.54 (0.03) | 2.14 | 1,21 | .16 | .50 |
| Lateral orbitofrontal | 2.60 (0.04) | 2.52 (0.03) | 2.51 | 1,21 | .13 | .62 |
| Pericalcarine | 1.51 (0.04) | 1.51 (0.03) | 0.00 | 1,21 | .99 | .00 |
|  |  |  |  |  |  |  |
| *Without alcohol dependent participants* | n=9 | n=14 |  |  |  |  |
|  |  |  |  |  |  |  |
| Whole brain | 2.47 (0.03) | 2.41 (0.02) | 2.16 | 1,16 | .16 | .55 |
| Superior frontal | 2.77 (0.05) | 2.75 (0.04) | 0.07 | 1,16 | .80 | .12 |
| Middle frontal | 2.46 (0.03) | 2.38 (0.02) | 4.80 | 1,16 | **.04** | .84 |
| Inferior frontal | 2.62 (0.05 | 2.56 (0.03) | 1.14 | 1,16 | .30 | .45 |
| Lateral orbitofrontal | 2.61 (0.05) | 2.53 (0.04) | 1.31 | 1,16 | .27 | .58 |
| Pericalcarine | 1.48 (0.04) | 1.53 (0.03) | 0.85 | 1,16 | .37 | .53 |
|  |  |  |  |  |  |  |
| *Without opioid using participants* | n=12 | n=15 |  |  |  |  |
|  |  |  |  |  |  |  |
| Whole brain | 2.47 (0.03) | 2.4 (0.02) | 3.20 | 1,20 | .09 | .58 |
| Superior frontal | 2.76 (0.05) | 2.73 (0.04) | 0.21 | 1,20 | .65 | .18 |
| Middle frontal | 2.46 (0.03) | 2.37 (0.02) | 6.13 | 1,20 | **.02** | .83 |
| Inferior frontal | 2.62 (0.04) | 2.53 (0.03) | 2.61 | 1,20 | .12 | .56 |
| Lateral orbitofrontal | 2.61 (0.04) | 2.51 (0.04) | 2.37 | 1,20 | .14 | .66 |
| Pericalcarine | 1.51 (0.04) | 1.52 (0.03) | 0.00 | 1,20 | .96 | .03 |
|  |  |  |  |  |  |  |

Estimated means (in mm) and standard errors. ANCOVA (only cocaine user groups, corrected for age, verbal IQ, ADHS-SR sum score, abstinence duration, and cumulative lifetime dose of cocaine). Significant p values are shown in bold.

***Table S3*.** Demographic data and drug use pattern of Study 2.

|  |  |  |  |  |  |  |
| --- | --- | --- | --- | --- | --- | --- |
|  | Controls  (n=38) | LowLevCU  (n=12) | HighLevCU  (n=17) | Value^a^ | df, df_err_ | p |
|  |  |  |  |  |  |  |
| Age (y) | 31.4 (7.6) | 31.2 (4.7) | 36.6 (7.9) | F=3.29 | 2,64 | **.04** |
| Sex (f/m) | 16/22 | 3/9 | 3/14 | x^2^=3.59 | 2 | .17 |
| Verbal IQ (MWT-B)^b,i^ | 109.0 (12.0) | 96.4 (7.0) **° | 107.6 (10.2) | F=6.31 | 2,64 | **.003** |
| Education (y) | 10.5 (1.5) | 10.3 (1.6) | 10.3 (1.5) | F=0.516 | 2,64 | .60 |
| Smoking (y/n)^c^ | 29/9 | 11/1 | 13/4 | x^2^=1.40 | 2 | .50 |
| BDI score^d^ | 2.3 (4.7) | 9.2 (7.0)** | 8.4 (7.8)** | F=9.340 | 2,64 | **<.001** |
| ADHD-SR score^e,i^ | 6.3 (5.6) | 15.6 (10.2)** | 16.0 (8.4)*** | F=14.00 | 2,64 | **<.001** |
|  |  |  |  |  |  |  |
| *Cocaine* |  |  |  |  |  |  |
| Times per week^f^ | - | 1.6 (2.0) | 1.2 (0.9) | T=0.77 | 27 | .45 |
| g per week^f^ | - | 1.9 (2.6) | 1.5 (1.4) | T=0.62 | 27 | .54 |
| Years of use | - | 7.8 (4.8) | 12.7 (6.6) | T=-2.21 | 27 | **.04** |
| Maximum dose (g/day) | - | 2.1 (1.3) | 2.5 (1.4) | T=-0.85 | 23 | .40 |
| Cumulative dose (g) | - | 1063 (1199) | 1744 (1600) | T=-1.25 | 27 | .22 |
| Last consumption (days)^g^ | - | 7.2 (7.7) | 10.5 (8.2) | T=-1.07 | 27 | .29 |
| Urine toxicology (neg/pos)^h^ | 38/0 | 6/6 | 8/9 | x^2^=0.02 | 1 | .88 |
| Average price paid for 1g (CHF)^j^ | - | 96.8 (22.4) | 93.2 (13.3) | T=0.53 | 26 | .60 |
| Hair analysis |  |  |  |  |  |  |
| Cocaine pg/mg | - | 26236 (24761) | 20974 (25661) | T=0.55 | 27 | .59 |
| Benzoylecgonine pg/mg | - | 10082 (12005) | 6395 (6641) | T=1.06 | 27 | .57 |
| Norcocaine pg/mg | - | 741 (801) | 546 (554) | T=0.78 | 27 | .45 |
| Levamisole pg/mg | - | 1867 (2633) | 9715 (12380) | T=-2.23 | 27 | **.02** |
| Levamisole-Cocaine-Ratio | - | 0.07 (0.1) | 0.52 (0.3) | T=-7.96 | 27 | **<.001** |
|  |  |  |  |  |  |  |
| *Alcohol* |  |  |  |  |  |  |
| g per week^f^ | 62.0 (68.6) | 149.8 (116.6) | 205.1 (178.8)*** | F=9.93 | 2,64 | **<.001** |
| Years of use | 11.8 (6.5) | 9.8 (5.5) | 17.1 (9.2)°* | F=4.42 | 2,61 | **.02** |
|  |  |  |  |  |  |  |
| *Nicotine* |  |  |  |  |  |  |
| Cigarettes per day^f^ | 4.6 (6.1) | 10.8 (8.1)* | 7.9 (10.1) | F=3.42 | 2,64 | **.04** |
| Years of use | 7.8 (7.0) | 10.8 (6.5) | 13.7 (10.8)* | F=3.24 | 2,64 | **.05** |
|  |  |  |  |  |  |  |
| *Cannabis* |  |  |  |  |  |  |
| g per week^f^ | 0.0 (0.1) | 1.5 (2.2) | 2.2 (6.3) | F=2.75 | 2,64 | .07 |
| Years of use | 3.3 (5.3) | 12.1 (7.5)** | 9.5 (9.7)*** | F=9.32 | 2,64 | **<.001** |
| Cumulative dose (g) | 35.8 (82.9) | 1925 (3329) | 3353 (4419)*** | F=10.02 | 2,63 | **<.001** |
| Last consumption (days)^g^ | 76 (59);n=11 | 21 (37)*;n=10 | 11 (11)**;n=11 | F=7.96 | 2,29 | **.002** |
| Urine toxicology (neg/pos)^h^ | 38/0 | 8/4 | 13/4 | x^2^=12.55 | 2 | **.002** |
|  |  |  |  |  |  |  |
| *Amphetamine* |  |  |  |  |  |  |
| g per week^f^ | 0.0 (0.0) | 0.1 (0.1)** | 0.0 (0.0) | F=5.20 | 2,64 | **.008** |
| Years of use | 0.1 (0.5) | 2.0 (2.3)* | 2.3 (3.8)** | F=7.41 | 2,63 | **.001** |
| Cumulative dose (g) | 0.2 (0.9) | 190.0 (336.4)***°° | 18.5 (39.9) | F=8.52 | 2,64 | **.001** |
| Last consumption (days)^g^ | 30 (0);n=1 | 42 (45);n=5 | 60 (57);n=5 | F=0.23 | 2,19 | .80 |
| Hair analysis pg/mg | 0 (0) | 138 (198) | 160 (425) | F=3.64 | 2,64 | **.03** |
|  |  |  |  |  |  |  |
| *MDMA* |  |  |  |  |  |  |
| Tablets per week^f^ | 0.0 (0.0) | 0.3 (0.6)* | 0.2 (0.6) | F=4.08 | 2,63 | **.02** |
| Years of use | 0.2 (1.1) | 3.2 (3.2) | 5.4 (6.5)*** | F=12.44 | 2,62 | **<.001** |
| Cumulative dose (tablets) | 0.8 (5.1) | 469.0 (1119.6)* | 247.9 (469.0) | F=4.24 | 2,62 | **.02** |
| Last consumption (days)^g^ | 91 (0);n=1 | 63 (57);n=9 | 26 (27);n=9 | F=2.10 | 2,16 | .16 |
| Hair analysis pg/mg | 2 (9) | 224 (267) | 2867 (8521) | F=2.77 | 2,64 | .07 |
|  |  |  |  |  |  |  |
| *Hallucinogens* |  |  |  |  |  |  |
| Cumulative dose (times) | 0.6 (1.6) | 2.0 (2.7) | 10.9 (23.3)* | F=4.50 | 2,61 | **.02** |
|  |  |  |  |  |  |  |

Means and standard deviations. Significant p values are shown in bold.

^a^ ANOVA (all groups; significant Sidak post-hoc test vs. control group: *p<.05; **p<.01; ***p<.001; vs. lowLevCU: °p<.05; °°p<.01); x² test (all groups/cocaine users only) for frequency data; Independent t-test (cocaine users only).

^b^ Verbal IQ was assessed by the Mehrfachwahl Wortschatz Intelligenztest[^3^](#_ENREF_3).

^c^ Smoking habits were assessed by the Fagerstroem Test of Nicotine Dependence[^4^](#_ENREF_4).

^d^ BDI, Beck Depression Inventory[^5^](#_ENREF_5).

^e^ ADHD-SR, ADHD self rating scale[^6^](#_ENREF_6).

^f^ Average use during the last 6 months.

^g^ Last consumption is averaged only for persons who used the drug in the last 6 months. In this case, sample size (n) is shown.

^h^ Cut-off values for cocaine = 150 ng/ml and for Tetrahydrocannabinol 50 ng/ml [^7^](#_ENREF_7).

^i^ For one LowLevCU and one highLevCU, the verbal IQ and ADHS-SR score were not available due to technical problems. For those participants, missing values were replaced with their group mean.

^j^ Price for 1 g cocaine in Swiss Francs paid by cocaine users (self-report). The quoted price is presumably below the real street price as some users paid reduced rates at intermediaries. Moreover, individuals who got the cocaine for free (e.g., as a gift) were excluded (n=1 lowLevCU).

***Table S4.*** Socioeconomic status (number of subjects and percent).

|  |  |  |  |  |  |  |
| --- | --- | --- | --- | --- | --- | --- |
|  | Study 1 | |  | Study 2 | |  |
|  |  | |  |  | |  |
|  | LowLevCU (n=26) | HighLevCU (n=49) |  | LowLevCU (n=12) | HighLevCU (n=17) |  |
|  |  |  |  |  |  |  |
| 0 - 15'000 CHF | 9 (34.6%) | 12 (24.5%) |  | 0 (0.0%) | 3 (17.6%) |  |
| 15'000 - 30'000 CHF | 8 (30.8%) | 8 (16.3%) |  | 1 (8.3%) | 1 (5.9%) |  |
| 30'000 - 60'000 CHF | 6 (23.1%) | 15 (30.6%) |  | 8 (66.7%) | 7 (41.2%) |  |
| 60'000 - 90'000 CHF | 2 (7.7%) | 9 (18.4%) |  | 3 (25.0%) | 1 (5.9%) |  |
| 90'000 - 120'000 CHF | 0 (0.0%) | 4 (8.2%) |  | 0 (0.0%) | 3 (17.6%) |  |
| 120'000 CHF and more | 1 (3.8%) | 1 (2.0%) |  | 0 (0.0%) | 2 (11.8%) |  |
|  |  |  |  |  |  |  |
| Fisher-Freeman-Halton Exact Test | F=6.03, p=.28 | |  | F=7.41, p=.13 | |  |
|  |  |  |  |  |  |  |

Participants were asked how much money they had available over the past year.

***Table S5.*** Neuropsychological test scores of controls vs. cocaine users in Study 1.

|  |  |  |  |  |  |  |
| --- | --- | --- | --- | --- | --- | --- |
|  | Controls (n=78) | Cocaine users  (n=75) | F | df, df_err_ | p | Cohen's d |
|  |  |  |  |  |  |  |
| Global Cognitive Index | -0.07 (0.06) | -0.58 (0.06) | 28.34 | 1,148 | **<.001** | .74 |
|  |  |  |  |  |  |  |
| *Neurocognitive domain scores* |  |  |  |  |  |  |
| Attention | -0.04 (0.10) | -0.58 (0.10) | 13.58 | 1,148 | **<.001** | .61 |
| Working memory | -0.11 (0.08) | -0.53 (0.08) | 13.32 | 1,148 | **<.001** | .54 |
| Declarative memory | -0.08 (0.10) | -0.72 (0.10) | 18.26 | 1,148 | **<.001** | .63 |
| Executive functions | -0.04 (0.09) | -0.51 (0.10) | 10.64 | 1,148 | **.001** | .54 |
|  |  |  |  |  |  |  |

Estimated means and standard errors. ANCOVA (all groups, corrected for age, verbal IQ, and ADHS-SR sum score). Significant p values are shown in bold. Significant Sidak post-hoc test vs. control group: *p<.05; **p<.01; ***p<.001. GCI and cognitive domain scores are z-transformed values.

***Table S6.*** Neuropsychological test scores of controls vs. low LCR cocaine users vs. high LCR cocaine users in Study 1.

|  |  |  |  |  |  |  | Cohen's d | | |
| --- | --- | --- | --- | --- | --- | --- | --- | --- | --- |
|  | Controls (n=78) | LowLevCU (n=26) | HighLevCU (n=49) | F | df, df_err_ | p | Controls  vs. LowLevCU | Controls  vs. HighLevCU | LowLevCU  vs. HighLevCU |
|  |  |  |  |  |  |  |  |  |  |
| Global Cognitive Index | -0.07 (0.06) | -0.47 (0.10)** | -0.65 (0.08)*** | 15.26 | 2,147 | **<.001** | .58 | .85 | .27 |
|  |  |  |  |  |  |  |  |  |  |
| *Neurocognitive domain scores* |  |  |  |  |  |  |  |  |  |
| Attention | -0.04 (0.10) | -0.56 (0.16)* | -0.59 (0.12)** | 6.76 | 2,147 | **.002** | .59 | .63 | .04 |
| Working memory | -0.11 (0.08) | -0.49 (0.12)* | -0.55 (0.10)** | 6.70 | 2,147 | **.002** | .49 | .57 | .08 |
| Declarative memory | -0.08 (0.10) | -0.53 (0.16) | -0.84 (0.12)*** | 10.45 | 2,147 | **<.001** | .45 | .76 | .31 |
| Executive functions | -0.04 (0.09) | -0.31 (0.15) | -0.64 (0.12)*** | 6.81 | 2,147 | **.001** | .32 | .70 | .38 |
|  |  |  |  |  |  |  |  |  |  |
| *Neuropsychological test scores* |  |  |  |  |  |  |  |  |  |
| *Attention* |  |  |  |  |  |  |  |  |  |
| RVP Discrimination performance A' | 0.916 (0.01) | 0.887 (0.01)* | 0.893 (0.01)* | 5.04 | 2,147 | **.008** | .62 | .49 | .13 |
| RVP Total hits | 18.21 (0.54) | 15.39 (0.89)* | 16.07 (0.70) | 4.47 | 2,147 | **.01** | .60 | .45 | .14 |
| RAVLT Supraspan trial 1 | 9.00 (0.24) | 8.56 (0.39) | 7.53 (0.31)** | 6.22 | 2,147 | **.003** | .19 | .63 | .44 |
|  |  |  |  |  |  |  |  |  |  |
| *Working memory* |  |  |  |  |  |  |  |  |  |
| LNST Score | 15.28 (0.33) | 14.01 (0.55) | 14.28 (0.43) | 2.48 | 2,147 | .09 | .41 | .32 | .09 |
| SWM Total errors | 20.10 (1.97) | 26.66 (3.23) | 29.05 (2.54)* | 3.72 | 2,147 | **.03** | .37 | .51 | .14 |
| PAL First trial memory score | 15.18 (0.38) | 14.05 (0.62) | 13.63 (0.49) | 3.00 | 2,147 | .05 | .32 | .43 | .12 |
|  |  |  |  |  |  |  |  |  |  |
| *Declarative memory* |  |  |  |  |  |  |  |  |  |
| RAVLT Learning performance (∑ trials 1-5) | 61.92 (0.89) | 58.35 (1.46) | 54.17 (1.15)*** | 12.59 | 2,147 | **<.001** | .39 | .84 | .46 |
| RAVLT Adj. recognition performance p(A) | 0.875 (0.01) | 0.838 (0.02) | 0.833 (0.02) | 1.90 | 2,147 | .15 | .31 | .36 | .04 |
| RAVLT Delayed recall trial 7 | 13.13 (0.27) | 12.12 (0.44) | 10.96 (0.35)*** | 10.78 | 2,147 | **<.001** | .39 | .84 | .45 |
| PAL Total errors adjusted | 11.38 (1.70) | 16.67 (2.79) | 18.47 (2.19) | 3.17 | 2,147 | **.05** | .34 | .46 | .12 |
| PAL Total trials adjusted | 8.66 (0.38) | 9.76 (0.61) | 10.33 (0.48)* | 3.46 | 2,147 | **.03** | .31 | .47 | .16 |
|  |  |  |  |  |  |  |  |  |  |
| *Executive functions* |  |  |  |  |  |  |  |  |  |
| IED Total errors adjusted | 29.95 (4.41) | 30.12 (7.21) | 41.56 (5.67) | 1.32 | 2,147 | .27 | .00 | .31 | .31 |
| IED Total trials adjusted | 104.00 (7.82) | 104.16 (12.79) | 126.86 (10.06) | 1.64 | 2,147 | .20 | .00 | .35 | .35 |
| SWM Strategy score | 32.47 (0.61) | 34.06 (1.00) | 34.44 (0.78) | 1.97 | 2,147 | .14 | .30 | .38 | .07 |
| RAVLT Recall consistency in % | 91.86 (1.09) | 87.29 (1.79) | 84.35 (1.41)*** | 8.10 | 2,147 | **<.001** | .45 | .74 | .29 |
|  |  |  |  |  |  |  |  |  |  |

Estimated means and standard errors. ANCOVA (all groups, corrected for age, verbal IQ, and ADHS-SR sum score). Significant p values are shown in bold. Significant Sidak post-hoc test vs. control group: *p<.05; **p<.01; ***p<.001. GCI and cognitive domain scores are z-transformed values.

***Table S7.*** Neuropsychological test scores Study 1. Cocaine user group comparison.

|  | LowLevCU (n=26) | HighLevCU (n=49) | F | df, df_err_ | p | Cohen's d |
| --- | --- | --- | --- | --- | --- | --- |
|  |  |  |  |  |  |  |
| Global Cognitive Index | -0.48 (0.12) | -0.76 (0.09) | 3.21 | 1,68 | .08 | .42 |
|  |  |  |  |  |  |  |
| *Neurocognitive domain scores* |  |  |  |  |  |  |
| Attention | -0.55 (0.18) | -0.66 (0.13) | .21 | 1,68 | .65 | .12 |
| Working memory | -0.62 (0.14) | -0.65 (0.10) | .03 | 1,68 | .86 | .04 |
| Declarative memory | -0.52 (0.20) | -0.98 (0.14) | 3.21 | 1,68 | .08 | .44 |
| Executive functions | -0.23 (0.18) | -0.74 (0.13) | 5.02 | 1,68 | **.03** | .55 |
|  |  |  |  |  |  |  |
| *Neuropsychological test scores* |  |  |  |  |  |  |
| *Attention* |  |  |  |  |  |  |
| RVP Discrimination performance A' | 0.886 (0.01) | 0.890 (0.01) | .09 | 1,68 | .76 | .08 |
| RVP Total hits | 15.35 (0.99) | 15.75 (0.70) | .10 | 1,68 | .75 | .08 |
| RAVLT Supraspan trial 1 | 8.65 (0.37) | 7.39 (0.26) | 7.15 | 1,68 | **.009** | .65 |
|  |  |  |  |  |  |  |
| *Working memory* |  |  |  |  |  |  |
| LNST Score | 13.5 (0.53) | 13.88 (0.37) | .32 | 1,68 | .57 | .14 |
| SWM Total errors | 27.53 (3.68) | 30.31 (2.61) | .35 | 1,68 | .56 | .15 |
| PAL First trial memory score | 13.45 (0.68) | 13.31 (0.48) | .03 | 1,68 | .87 | .04 |
|  |  |  |  |  |  |  |
| *Declarative memory* |  |  |  |  |  |  |
| RAVLT Learning performance (∑ trials 1-5) | 59.08 (1.75) | 53.04 (1.24) | 7.34 | 1,68 | **.009** | .63 |
| RAVLT Adj. recognition performance p(A) | 0.839 (0.03) | 0.825 (0.02) | .18 | 1,68 | .67 | .11 |
| RAVLT Delayed recall trial 7 | 12.32 (0.53) | 10.67 (0.37) | 6.02 | 1,68 | **.02** | .61 |
| PAL Total errors adjusted | 17.7 (3.81) | 20.00 (2.69) | .22 | 1,68 | .64 | .13 |
| PAL Total trials adjusted | 9.99 (0.78) | 10.76 (0.55) | .60 | 1,68 | .44 | .20 |
|  |  |  |  |  |  |  |
| *Executive functions* |  |  |  |  |  |  |
| IED Total errors adjusted | 25.79 (8.17) | 43.46 (5.79) | 2.87 | 1,68 | .09 | .44 |
| IED Total trials adjusted | 96.19 (14.35) | 130.12 (10.16) | 3.43 | 1,68 | .07 | .48 |
| SWM Strategy score | 34.27 (0.97) | 34.63 (0.69) | .08 | 1,68 | .77 | .08 |
| RAVLT Recall consistency in % | 87.86 (2.37) | 82.86 (1.68) | 2.75 | 1,68 | .10 | .41 |
|  |  |  |  |  |  |  |

Estimated means and standard errors. ANCOVA (only cocaine user groups, corrected for age, verbal IQ, ADHS-SR sum score, abstinence duration, and cumulative lifetime dose of cocaine). Significant p values are shown in bold. GCI and cognitive domain scores are z-transformed values.

## Figure S1


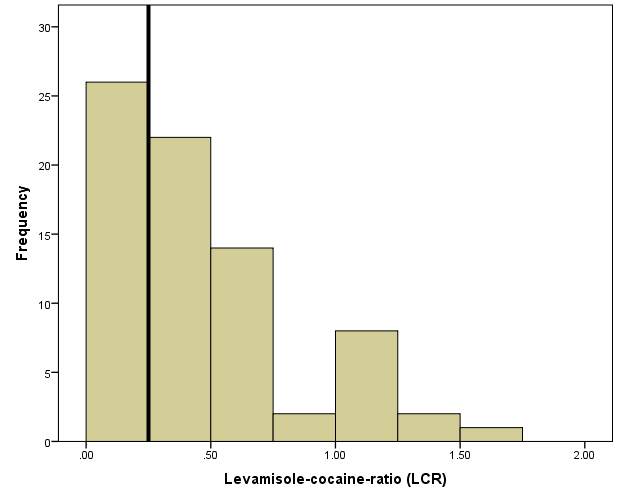


Levamisole-cocaine-ratio frequency chart (n=75). The bold black line represents the group assignment LCR-cutoff of 25%.

## Figure S2


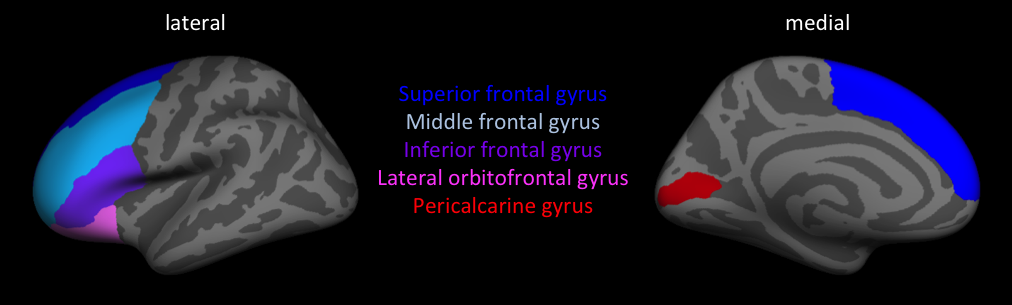


Regions of interest (ROI) included in this study projected to the inflated surface of FreeSurfer’s average template. From these regions, cortical thickness was extracted. Left hemisphere is indicated for visualization, however, cortical thickness within the same ROIs were also extracted from the right hemisphere.

## Figure S3

Estimated means and standard errors in lowLevCU (n=26) and highLevCU (n=49). ANCOVA (only cocaine user groups, corrected for age, verbal IQ, ADHS-SR sum score, abstinence duration, and cumulative lifetime dose of cocaine). Significant Sidak post-hoc test: *p<.05; Cohen’s d.

## Figure S4


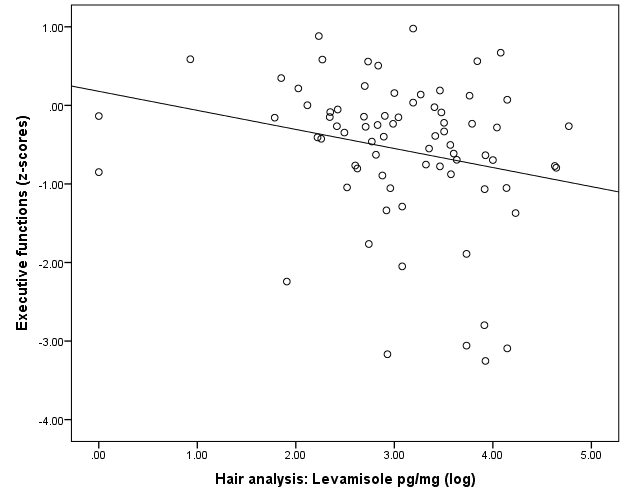


Pearson’s product-moment correlations between executive functions z-scores and log-transformed levamisole concentrations in hair (the constant 1was added because the data of two CU contained 0 values) in a combined CU sample (n=75, r= -.23, p<.05, one-tailed).

## Figure S5


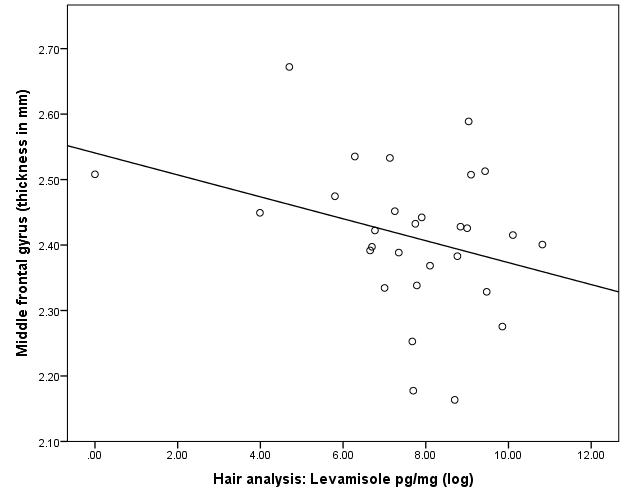


Pearson’s product-moment correlations between the middle frontal gyrus thickness (in mm) and log-transformed levamisole concentrations in hair (the constant 1was added because the data of one CU contained a 0 value) in a combined CU sample (n=29, r= -.32, p<.05, one-tailed).

## References

1. Fischl B, Dale AM. Measuring the thickness of the human cerebral cortex from magnetic resonance images. *Proc Natl Acad Sci U S A* 2000; **97**(20)**:** 11050-11055.

2. Kuperberg GR, Broome MR, McGuire PK, David AS, Eddy M, Ozawa F *et al.* Regionally localized thinning of the cerebral cortex in schizophrenia. *Arch Gen Psychiatry* 2003; **60**(9)**:** 878-888.

3. Lehrl S. *Mehrfachwahl-Wortschatz-Intelligenztest MWT-B. Fünfte Auflage.* Spitta Verlag: Balingen, 2005.

4. Heatherton TF, Kozlowski LT, Frecker RC, Fagerstrom KO. The Fagerstrom Test for Nicotine Dependence: a revision of the Fagerstrom Tolerance Questionnaire. *Br J Addict* 1991; **86**(9)**:** 1119-1127.

5. Hautzinger M, Bailer M, Worall H, Keller F. *Beck-Depressions-Inventar (BDI). Bearbeitung der deutschen Ausgabe. Testhandbuch. (Beck Depression Inventory. Test manual. 2nd ed.)*. Huber: Bern, Göttingen, Toronto, Seattle, 1994.

6. Rosler M, Retz W, Retz-Junginger P, Thome J, Supprian T, Nissen T *et al.* [Tools for the diagnosis of attention-deficit/hyperactivity disorder in adults. Self-rating behaviour questionnaire and diagnostic checklist]. *Nervenarzt* 2004; **75**(9)**:** 888-895.

7. Substance Abuse and Mental Health Services Administration. Mandatory Guidelines for Federal Workplace Drug Testing Programs. *Fed Regist* 2008; **73**(228)**:** 71858-71907.
